# COVID-19 and One-Carbon Metabolism

**DOI:** 10.3390/ijms23084181

**Published:** 2022-04-10

**Authors:** Joanna Perła-Kaján, Hieronim Jakubowski

**Affiliations:** 1 Department of Biochemistry and Biotechnology, University of Life Sciences, 60-632 Poznań, Poland; kajan@up.poznan.pl; 2 Department of Microbiology, Biochemistry and Molecular Genetics, Rutgers-New Jersey Medical School, Newark, NJ 07103, USA

**Keywords:** folate, purine biosynthesis, methionine, S-adenosylmethionine, S-adenosylhomocysteine, homocysteine, cysteine, glutathione, choline, methionine sulfoxide

## Abstract

Dysregulation of one-carbon metabolism affects a wide range of biological processes and is associated with a number of diseases, including cardiovascular disease, dementia, neural tube defects, and cancer. Accumulating evidence suggests that one-carbon metabolism plays an important role in COVID-19. The symptoms of long COVID-19 are similar to those presented by subjects suffering from vitamin B_12_ deficiency (pernicious anemia). The metabolism of a cell infected by the SARS-CoV-2 virus is reshaped to fulfill the need for massive viral RNA synthesis, which requires de novo purine biosynthesis involving folate and one-carbon metabolism. Many aspects of host sulfur amino acid metabolism, particularly glutathione metabolism underlying antioxidant defenses, are also taken over by the SARS-CoV-2 virus. The purpose of this review is to summarize recent findings related to one-carbon metabolism and sulfur metabolites in COVID-19 and discuss how they inform strategies to combat the disease.

## 1. Introduction

Severe acute respiratory syndrome coronavirus 2 (SARS-CoV-2) is an enveloped (+)ssRNA virus, in which the infecting RNA acts as a messenger RNA (mRNA). After entering the host cell, SARS-CoV-2 is replicated. This process involves the translation of viral mRNA by cellular ribosomes to produce the viral replicative enzymes, which generate new RNA genomes and the mRNAs for the synthesis of the components necessary to assemble the new viral particles [1].

SARS-CoV-2 is the cause of a global pandemic of coronavirus disease of 2019 (COVID-19). On 31 December 2019, the World Health Organization’s (WHO) country office in China registered cases of ‘viral pneumonia’ in Wuhan. A month later, on 30 January 2020, WHO’s Director-General declared the novel coronavirus outbreak a public health emergency of international concern. On 11 March 2020, WHO made the assessment that COVID-19 could be characterized as a pandemic. Globally, as of 4 April 2022, there have been 494 million confirmed cases of COVID-19, including 6.15 million deaths. The highest number of COVID-19 cases, some 80.1 million, were in the United States, and included 0.98 million deaths. The scale of health and economical threats caused by the pandemic outbreak urged many scientific groups to research the mechanisms triggered by the virus to allow treatment and vaccination. Several SARS-CoV-2 variants have emerged since the first identified strain, apparently with higher transmissibility/virulence and immune escape capabilities.

Interestingly, COVID-19 patients present a diverse severity of clinical manifestations, ranging from no symptoms to death. Of the total COVID-19 cases, about 80% are either asymptomatic or experience a mild course of the disease, while about 14% develop severe symptoms, such as pneumonia, and about 5% present critical symptoms, such as septic shock, respiratory failure, or multi organ failure, and finally about 2% of the subjects die. In general, the worse course of the disease is associated with old age and comorbidities, especially chronic obstructive lung disease, obesity, diabetes mellitus, cardiovascular disease and hypertension [2].

To complicate matters, in a considerable fraction of patients, SARS-CoV-2 infection is followed by a complication called long COVID-19, which can last for months and has diverse symptoms such as fatigue, headache, ‘brain fog’, anosmia, myalgia, dizziness, breathlessness, palpitations, and gastrointestinal problems. The prevalence of long COVID-19 is based on ten reporting studies, and ranged from 4.7% to 80%. The frequency of most prevalent long COVID-19 symptoms that may last from weeks to months after acute infection was as follows: chest pain—up to 89%, fatigue—up to 65%, dyspnea—up to 61%, cough and sputum production—up to 59%, cognitive and memory impairment—up to 57.1%, arthralgia—up to 54.7%, sleep disorders—up to 53%, and myalgia—up to 50.6%. The list of other signs and symptoms of long COVID-19 with lower frequency contains over thirty records, all of which are listed with reporting studies [3].

The progression of COVID-19 can be divided into three overlapping phases: early infection, pulmonary phase and hyperinflammation [4]. As the lung parenchyma is targeted by the virus, the organism activates innate immune response, and the following effects may be triggered: inflammation, damage to the vessel walls, vasodilation and endothelial permeability, pulmonary restriction, hypoxemia, and increased cardiovascular stress. Respiratory failure, if present, together with viral infiltration into myocardial tissue and cardiac inflammation leads to cardiac injury [4]. Kumar et al. [2] have described the COVID-19 mechanisms in the human body, including symptomatology, virus–host interactions, and host factors affecting transmissibility, severity and outcomes (age, sex and comorbidities) as well as organ-specific pathologies ongoing in the respiratory, cardiovascular, renal, digestive, and nervous systems during SARS-CoV-2 infection [2].

A summary of over twenty proteomic studies on plasma and serum of COVID-19 patients revealed three deregulated KEGG pathways: complement and coagulation cascades, cytokine-cytokine receptor interaction and cholesterol metabolism [5]. Elevations of inflammation biomarkers such as IL (interleukin)-6, IL-2, IL-7, TNF (tumor necrosis factor), MCP (monocyte chemoattractant protein)-1, MIP (macrophage inflammatory protein)-1, G-CSF (granulocyte-colony stimulating factor), CRP (C-reactive protein), procalcitonin and ferritin are associated with increased mortality [4] and higher disease severity [6]. The results of a cohort study with 84 patients diagnosed with COVID-19 from Wuhan, China, demonstrated that the level of cardiac enzymes, as well as the abnormalities in the ECG, correlate positively with the level of inflammation values, in particular CRP and procalcitonin [7]. As shown recently, multi organ failure in patients with severe COVID-19 complication is caused by systemic vasculitis and cytokine mediated coagulation. Other identified biomarkers are hematological (lymphocyte count, neutrophil count, neutrophil-lymphocyte ratio), erythrocyte sedimentation rate, D-dimer, troponin, creatine kinase, and aspartate aminotransferase. Homocysteine (Hcy) and angiotensin II were also suggested to play significant roles [8].

**Figure 1:**
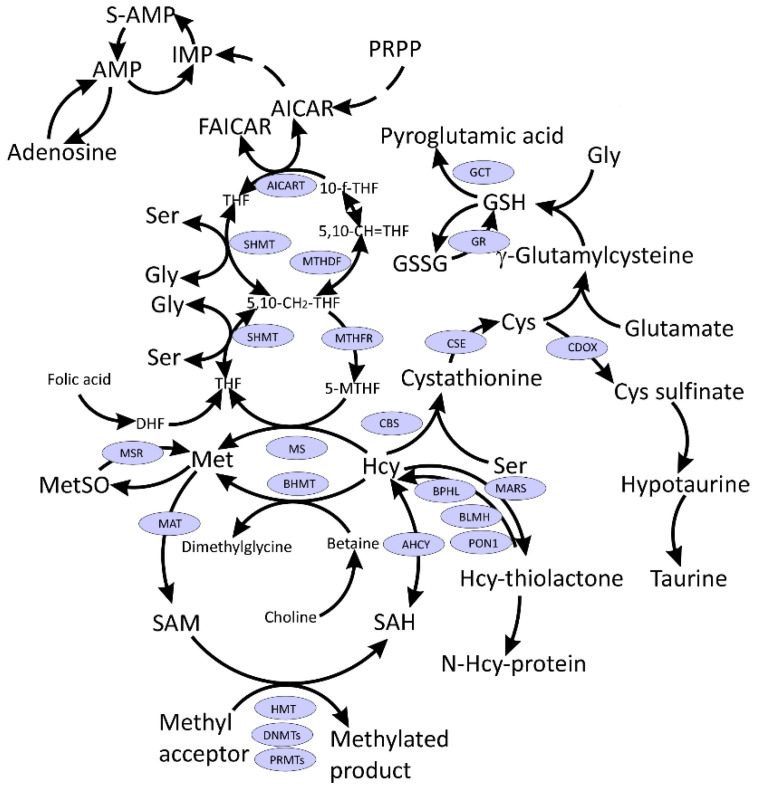
Interconnections between metabolism of folate, one-carbon, and sulfur compounds. Indicated metabolites are discussed in the text. CysGly, a product of GSH catabolism, affected in COVID-19 and discussed in the text, is not shown.

The symptoms of long COVID-19 are similar to those presented by subjects suffering from pernicious anemia (a condition caused by vitamin B_12_ deficiency), where methylation status is compromised [9,10]. Vitamin B_12_ is a cofactor of the key one-carbon metabolism enzyme—vitamin B_12_-dependent methionine (Met) synthase (MS) that remethylates Hcy to Met and links Met and folate cycles (Figure 1). MS generates Met, which then is used for the production of S-adenosylmethionine (SAM), a universal methyl donor, for a variety of acceptors, many of which participate in epigenetic regulation of gene expression. Moreover, one-carbon metabolism supports multiple physiological processes, such as biosynthesis of purines and thymidine, amino acid homeostasis of glycine (Gly), serine, and Met, and underlies antioxidant defense via glutathione (GSH) (Figure 1). Additionally, one-carbon metabolism is also important in the generation of energy via adenosine triphosphate (ATP) production in the mitochondria [9,10,11].

Accumulating evidence suggests that one-carbon metabolism plays an important role in COVID-19. The purpose of this review is to summarize recent findings related to sulfur amino acids and one-carbon metabolism in COVID-19 and discuss how they inform strategies to combat the disease.

## 2. SARS-CoV-2 Hijacks Host Folate and One-Carbon Metabolism

Metabolism of a cell infected by a virus is reshaped to fulfill the viral needs for its successful replication. Under viral infection, anabolic reactions are dominant and there is an upregulation of the ingestion of an extracellular carbon source (e.g., glucose or glutamine), which is used for lipogenesis and nucleotide synthesis, both of which are crucial for viral replication [1].

Several amino acids and their derivatives play important roles in one-carbon metabolism (Figure 1). Activated Met in the form of SAM is a universal methyl donor, Hcy is an intermediate in Cys synthesis, which in turn is needed for glutathione synthesis responsible for redox homeostasis. Ser is the precursor for the biosynthesis of several amino acids including Gly and Cys and participates in folate metabolism by donating one-carbon units for the biosynthesis of purines and pyrimidines.

Insight into how SARS-CoV-2 remodels the host metabolism to support the virus replication has been provided by ex vivo studies using the African Green Monkey Vero E6 cells [12]. Upon infection with SARS-CoV-2 isolate, the cells rapidly produced viral genomic RNA and nucleocapsid protein by 8 h post-infection, at which time the induction of antiviral genes, NF-ĸB targets, and ER stress response was observed. These changes were accompanied by a global decrease in host mRNAs with no changes in levels of mRNAs encoding metabolic enzymes. The changes in the patterns of mitochondrial DNA expression indicated ATP depletion. One of the most striking changes was the elevation of *de novo* purine synthesis intermediates (PPRP, FGAR, AIR, SAICAR) (Figure 1) in the SARS-CoV-2-infected cells and the reduction of intracellular folate levels (Table 1). Other metabolites participating in one-carbon metabolism and sulfur amino acid metabolism were also affected by the SARS-CoV-2 infection (Supplementary Data 4 in Ref. [12]). For example, intracellular Met, cystathionine, pyridoxine, betaine, serine, Gly, 5-oxoproline (pyroglutamate), and cysteine-glutathione disulfide levels were attenuated, while reduced glutathione levels were elevated. Intracellular SAM, SAH, cysteine (Cys), oxidized glutathione (GSSG) levels were not affected in the SARS-CoV-2-infected cells. These findings suggest that SARS-CoV-2 hijacks folate and one-carbon metabolism to meet the demands for viral replication [12]. As shown in Table S1 and discussed below, these metabolites were also affected by SARS-CoV-2 infection in vivo in COVID-19 patients.

## 3. S-Adenosylmethionine and Methylation Index

The SAM/SAH ratio, known as the methylation index, may be affected by SARS-CoV-2 infection. As mentioned earlier, SAM is required for capping of the viral RNA. The RNA cap (m7GpppN-RNA) is composed of a 7-methylguanosine (m7G) linked to the 5′-nucleoside (N) of the RNA chain through a triphosphate bridge (ppp). The cap structure is methylated at the N7 position of the guanosine by the C-terminal (guanine-N7)-methyltransferase (N7-MTase) domain of nonstructural protein 14 (Nsp14), forming cap-0 (m7GpppN-RNA), using SAM as a methyl donor [13,14]. The second methylation reaction during cap synthesis is catalyzed by SAM-dependent Nsp16 methyltransferase, which adds the methyl group on the ribose 2′-O position of the first transcribed nucleotide to form cap-1 (m7GpppNm-RNA). The RNA final cap has several important biological roles in viruses as it is critical for the stability of mRNAs, both for their translation and to evade the host immune response [14].

It has been hypothesized that SARS-CoV-2 infection may lead to SAM depletion in patients suffering long-term consequences of COVID-19. However, although SAM has not been quantified in long COVID-19, this hypothesis doesn’t seem to hold much water because several studies showed significant increases or no changes in plasma SAM levels in COVID-19 cases (Table S1). Moreover, SAH levels are either elevated [15,16], attenuated [17] or do not change in COVID-19 [18]. For example, a study on fifty-six COVID-19 patients admitted to the hospital between September and December 2020 in Moscow, Russia, has shown that an elevated SAM level and SAM/SAH and SAM/glutathione ratios have been associated with an increased risk of severe lung injury. Furthermore, an elevated SAM concentration and SAM/SAH and SAM/GSF ratios have been associated with an increased risk of lung damage [18]. Metabolomic analyses have revealed that SAM was significantly elevated in critical cases of COVID-19 [15] and those with a fatal outcome [19] as compared to control, mild and moderate cases of COVID-19 [15] (Table S1). Even though SAM was highest among severe COVID-19 patients, it was associated with a favorable prognosis. On the other hand, while there was no association between dimethylglycine, a by-product of Hcy remethylation to Met by a betaine-dependent enzyme BHMT (Figure 1), and COVID-19 stage, dimethylglycine was significantly lower in patients with an unfavorable progression of COVID-19 [15].

## 4. Methionine and Methionine Sulfoxide

The results of metabolomic studies on Met are contradictory, showing upregulation [16], downregulation [20,21], or no change in Met levels [17,22] in COVID-19 cases vs. healthy controls (Table S1). The direction of changes in Met level depends on compared groups, i.e., there is a tendency to higher Met levels in critical COVID-19 patients vs. healthy controls, but Met was lower in mild COVID-19 patients vs. healthy controls and there was no change in Met levels in patients with moderate COVID-19 vs. controls [15].

Metabolomic analyses of blood samples from COVID-19 patients and COVID-19-negative subjects revealed the significant impact of SARS-CoV-2 infection on serum Met sulfoxide, which consistently showed increased levels in four independent studies, suggesting increased oxidant stress [16,17,23,24] (Table S1).

## 5. Glutathione and Related Metabolites

COVID-19 is associated with disrupted redox homeostasis and reactive oxygen species (ROS) accumulation. In May 2020, Polonikov published a hypothesis which stated that [25]: “glutathione deficiency is the most plausible explanation for serious manifestation and death in COVID-19 patients”. Glutathione (GSH) depletion has been observed in diseases that increase the risk of COVID-19 [26]. GSH, being the main antioxidant agent, was suggested to be essential for counterbalancing the inflammation observed in SARS-CoV-2 infected patients (reviewed in Ref. [27]).

The glutathione hypothesis of COVID-19 appears to be supported by available data (Table S1). Indeed, GSH levels are consistently decreased in COVID-19 patients [17,18,28,29]. For example, a study on fifty-nine COVID-19 patients admitted to the hospital between August and November 2020 in Moscow, Russia [29], found that the levels of total GSH (tGSH) were significantly lower in moderate and severe COVID-19 patients compared with mildly affected subjects, while reduced CysGly (rCG) was significantly decreased in patients with higher degrees of lung damage based on percentage of lobar involvement (>26%) as compared to subjects with a lower degree of lung damage (0–25% of lobar involvement) (Table 2). tGSH and rCG were suggested to be risk markers for the severity of COVID-19 and lung damage in patients [29]. In addition, a negative correlation between rGSH and advance oxidation protein products in patients with high lung damage was observed [29]. A similar study involving fifty-six COVID-19 patients admitted to the hospital between September and December 2020 in Moscow, Russia [18], found lower GSH concentration in patients with a higher degree of lung damage (>50% of lobar involvement) as compared to patients with a lower degree of lung injury (<25% of lobar involvement). There also has been a significant increase in SAM level and SAM/GSH ratios, and a tendency to higher Hcy levels in subjects with more injured lungs [18] (Table 2).

**Figure 2:**
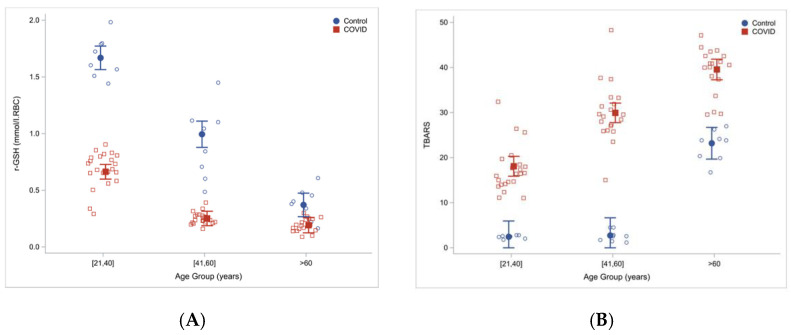
Reduced glutathione concentrations (**A**) and increased oxidative stress (TBARS) (**B**) in COVID-19 patients and uninfected controls stratified by age. Reproduced from Ref. [28] with permission.

Another study involving sixty COVID-19 patients hospitalized in Houston, TX and twenty-four uninfected controls, found that total and reduced red blood cells GSH were significantly lower in COVID-19 patients then in controls. At the same time, measures of lipid peroxidation, indicating oxidative stress (TBARS and F2-isoprostanes), were significantly elevated in COVID-19 patients and increased with age [28]. Severe GSH deficiency and oxidative damage also occur in young COVID-19 patients, and the magnitude of these defects in COVID-19 increased with age (Figure 2).

Cys levels were elevated in plasma [24], decreased in serum [21] and plasma [20] or unchanged in serum [22] and plasma [15]. Cystine, the oxidized disulfide form of Cys, was elevated in serum and plasma in several studies [16,21,22] and downregulated with IL-6 increase [23]. In another study, Cys was elevated in moderate COVID-19 patients vs. controls; however, analysis of critical and mild COVID-19 patients showed no changes in Cys levels vs. control group [15]. Cystathionine, an intermediate in the transsulfuration pathway (Figure 1), was either upregulated in plasma [30] and serum [17] or downregulated in the plasma of COVID-19 patients [24]. CysGly, a product of GSH metabolism, was shown to decrease in patients with higher degree of lung damage as diagnosed by computer tomography [29] or increase in plasma of COVID-19 patients as compared with controls [16].

Gly, which participates in GSH biosynthesis, was found to either increase [22], decrease [15,23], or was unchanged [16,17,21,31] in COVID-19 patients. In addition, pyroglutamate, a metabolite that forms from γ-glutamyl-Cys (glutathione precursor) (Figure 1) when Gly is limiting, was shown to be downregulated in COVID-19 patients [17] (Table S1).

## 6. Homocysteine

Hyperhomocysteinemia (HHcy) is linked to more than a hundred diseases and outcomes and has numerous detrimental effects, including neurotoxic, neuroinflammatory, neurodegenerative, proatherogenic, prothrombotic, and prooxidative. HHcy, a risk factor for cardiovascular disease, may be caused by the C667 > T MTHFR mutation, but also by reduced levels of folic acid and other B-vitamins [32]. The association of cardiovascular damage with COVID-19 and the fact that ischemic heart disease and hypertension are among the most frequent pre-existing comorbidities in COVID-19 patients, led to a suggestion that plasma Hcy can be “a potential marker for severe disease in SARS-CoV-2 patients” [33].

A recent study found that Hcy was significantly elevated in mild (12.73 μM, *n* = 74) and severe (15.62 μM, *n* = 43) COVID-19 cases compared with healthy controls (8.17 μM, *n* = 34). In ROC analysis, Hcy cutoff values of 9.18 μM (sensitivity 76.7%, specificity 76.5%, AUC = 0.951) and 10.3 μM (sensitivity 81.8%, specificity 82.4%, AUC = 1.000) identified mild and severe COVID-19 cases, respectively [34]. In multivariate logistic regression analysis, Hcy and two other variables (monocyte/lymphocyte ratio and fibrin D-dimer) were associated with mild and severe COVID-10. Other relevant variables that are known to be associated with Hcy levels, such as age and sex, have not been included in these analyses. Because there were significant differences in age and sex between COVID-19 cases (mild: 57.96 years, 52.5% male; severe: 71.22 years, 62.8% male) and healthy controls (32.65 years, 20.6% [34], it is not clear whether the differences in Hcy levels were due to COVID-19 or age/gender differences between the groups.

It has also been suggested that *MTHFR C677T* polymorphism may provide explanation for differences in geographical and gender distribution in COVID-19 severity [35] (November 2020). This suggestion was supported by a study [36] of genomic data available in the Genome Aggregation Database (genomAD), and the COVID-19 prevalence and mortality data (as of 27 August 2020), which identified a strong correlation between the prevalence of the *MTHFR C677T* polymorphism and COVID-19 incidence and mortality worldwide. The prevalence of *MTHFR 677T* allele in the Latino and European (non-Finnish) populations, and the incidence and mortality for COVID-19 were higher than reported for most other ethnic groups globally [37]. It has also been suggested that B-vitamins should be used to lower HHcy coexisting with COVID-19 [36]. Consequently to the proposed involvement of HHcy in COVID-19, it was suggested that Hcy may contribute to severe COVID-19 by interfering with G-protein-coupled receptors (GPCRs) (AT1R, B2 and CXCR6), by their upregulation, being an alternative agonist and inducing their heteromerization [38]. However, most of these proposals lack an experimental support.

In a multicenter retrospective study on 313 COVID-19 patients hospitalized between April and September 2020, Hcy was significantly elevated in non-survivors compared with survivors and the authors stated that Hcy was a predictor of severe disease progression; however, this is not shown in the results [39,40]. In another study of hospitalized patients with mild COVID-19 (*n* = 273), Hcy among other variables (age, monocyte-lymphocyte ratio, and period from onset to admission) could predict imaging progression on chest CT at first week from COVID-19 patients [41].

Hcy may be linked to COVID-19 by contributing to coagulopathy and thrombosis, conditions that often develop in SARS-CoV-2 infected patients. Examination of genome-wide associations and tissue-specific gene expression, aimed at elucidating the genetic basis of thrombosis in COVID-19 has led to annotation of various SNPs with five ancestral terms: pulmonary embolism, venous thromboembolism, vascular diseases, cerebrovascular disorders, and stroke. The gene-gene interaction network revealed three clusters that contained hallmark genes for D-dimer/fibrinogen levels, Hcy levels, and arterial/venous thromboembolism with F2 and F5 acting as connecting nodes. Based on these analyses it was suggested that genotyping COVID-19 patients for SNPs examined in this study would help to identify individuals at the greatest risk of complications linked to thrombosis [42].

However, examination of data from several studies shows inconsistent relationships between Hcy and COVID-19 (Table S1). Specifically, Hcy has been found to be *higher* in COVID-19 cases vs. healthy controls in three studies [33,39,41] and *lower* in COVID-19 cases vs. healthy controls in two other studies [17,23]. Other studies reported *no change* in Hcy levels between COVID-19 cases and controls [18,22,24].

## 7. Other One-Carbon Metabolites

Metabolomic analyses showed that serum serine was enriched in COVID-19 and COVID-19-like patients vs. healthy controls [31], while in other studies serine was found to decrease in more severe COVID-19 cases [20,23] or did not change between patients with different disease severity [16,17,21]. Serine was decreased in critical COVID-19 patients vs. controls [15]. Choline and its derivatives were downregulated in COVID-19 patients [43] (Table S1), suggesting that they might benefit from choline supplementation.

In COVID-19 children with mild symptoms vs. healthy children, methylmalonic acid MMA), Met sulfoxide and Cys increased, while choline, dimethylglycine, and methylcysteine decreased [24] (Table S1). MMA was upregulated 3.2-fold compared to healthy children. In contrast to COVID-19 children, MMA was reduced in COVID-19 adults to 3.1% of its levels in healthy adults. MMA is produced during catabolism of some amino acids (e.g., valine, isoleucine) and lipids (cholesterol, fatty acids), and is further metabolized by a vitamin B_12_-dependent enzyme malonyl-CoA mutase to succinic acid, which is a substrate for the TCA cycle. Interestingly, MMA was shown to inhibit replication of the mouse hepatitis virus (MHV), a well-known surrogate for SARS-CoV-2 in rat lung epithelial cells L2. Moreover, MMA reduced expression of inflammatory cytokines IL-6, TNF-α, and TGF-β in MHV-infected L2 cells [24]. Taken together, these findings suggest an antiviral and anti-inflammatory role of MMA in COVID-19-children, which can contribute to a mild course of SARS-CoV-2 infection in children compared with adults.

AICAR (5-Aminoimidazole-4-carboxamide ribonucleotide) was shown to be upregulated in COVID-19 patients [17], consistent with a suggestion that SARS-CoV-2 hijacks the host’s folate and one-carbon metabolism for viral RNA synthesis [12]. Adenosine, another metabolite generated during hydrolysis of SAH (Figure 1), was shown to be upregulated in COVID-19 patients [16,23], or not affected as a result of SARS-CoV-2 infection [17]. Taurine was either down-regulated [17,23] or did not change between studied groups [16]. Hypotaurine was upregulated in COVID-19 cases, as was cysteinyl-*S*-sulphate [16] (Table S1).

## 8. SARS-CoV-2 in Relation to RAS

Apart from basic functions such as regulation of blood pressure and vasoconstriction, renin-angiotensin system (RAS) may have pro-inflammatory and pro-fibrotic effects. RAS is implicated in the pathogenesis of hypertension, diabetes mellitus and obesity, which all increase the risk of a severe course of COVID-19. The binding of angiotensin II to its receptors mediates the generation of free radicals and causes mitochondrial dysfunction and tissue damage [44]. As recently discovered, RAS has a direct link to one-carbon metabolism, through Hcy (discussed below), which was found to activate one of the RAS receptors [22]. SARS-CoV-2 infection perturbs RAS and energy metabolism [45].

One of the important RAS players is angiotensin-converting enzyme 2 (ACE2) which converts angiotensin I (Ang I) to angiotensin 1-9 and angiotensin II (Ang II) to angiotensin 1-7. Membrane bound ACE2 (mACE2) is a zinc-containing a single-pass type I membrane protein located on the surface of intestinal enterocytes, renal tubular cells and other cells. The extracellular domain of mACE2 can be cleaved from the transmembrane domain by an enzyme referred to us ADAM17, during the protective phase of RAS. The resulting cleaved protein is known as soluble ACE2 (sACE2). sACE is released into the bloodstream where it cleaves Ang II into angiotensin 1-7 which binds to MasR receptors creating localized vasodilation and hence decreasing blood pressure.

On the other hand, angiotensin-converting enzyme (ACE), cleaves Ang I into the vasoconstricting Ang II that causes a cascade of hormonal reactions, which is part of the body’s harmful phase of RAS, leading to an increase in the blood pressure. Hence, sACE2 acts as a counterbalance to the ACE, degrading Ang II into angiotensin 1-7, and lowering blood pressure. A balance between ACE and ACE2 is curtailed for Ang II levels.

Another possible link between RAS and Hcy may involve homocysteinylation of ACE. Modification with Hcy and/or Hcy-thiolactone increases ACE activity leading to endothelial dysfunction [46].

The proper function of RAS depends on a balance between the two axis: the ACE-Ang II-AT1R axis, which has numerous detrimental effects, like vasoconstriction, inflammation, oxidative stress and fibrosis, and the ACE2-Ang 1-7-Mas receptor axis, which displays protective functions, like vasodilation, decreased fibrosis, and decreased inflammation. One of the cellular functions of ACE2 is cleavage of angiotensin II (Ang II) to angiotensin 1-7, and hence protecting against pathogenic effects of ACE-Ang II-AT1R axis of RAS. Obese subjects have increased levels of Ang II and proinflammatory cytokines (TNFα, IL-6, MCP-1) [47]. Tumor necrosis factor-alpha convertase (ADAM17) regulates Ang II and proinflammatory cytokines and mediates regulated ectodomain shedding of ACE2 [48]. The endocytosis of SARS-CoV-2 increases the activity of ADAM17, which in turn leads to the ACE2 shedding [49].

Also, spike protein of SARS-CoV was found to down-regulate ACE2 ectodomain expression [30]. These effects lead to disruption of balance between detrimental ACE-Ang II-AT1R axis and protective ACE2-Ang 1-7-Mas receptor of RAS and shift the RAS effects towards induction of inflammation and ROS production [50].

Patients with comorbidities (hypertension, cardiovascular disease, renal insufficiency, autoimmune disease) associated with severe COVID-19 have increased expression of ACE2 is their lungs [51]. mACE2 also serves as the entry point into cells for some coronaviruses, including HCoV-NL63, SARS-CoV, and SARS-CoV-2 [5]. It has been suggested that subjects with comorbidities may have higher chances of developing severe COVID-19, since ACE2 facilitates SARS-CoV-2 entry into the lung cells [51].

ACE2 is a functional receptor for coronaviruses [52], including SARS-CoV-2, which engages ACE2 for host cell entry through membrane fusion and endocytosis [53]. ACE2 binds to the receptor-binding domain (RBD) of SARS-CoV-2 spike (S) protein. Furthermore, to enter into the host cell, the priming of the viral spike protein (S) is considered essential for its fusion to the host cell membrane, which involves cleavage of the “S” protein by serine proteases called transmembrane serine protease 2 (TMPRSS2) or by cathepsin B or L (CTS-B or -L) and furin present in the host cell membrane [2]. The entry of SARS-CoV-2 to the cell is facilitated by a host factor neuropilin-1 facilitates cell entry and infectivity [54,55].

A molecular dynamic study has suggested that binding of the COVID-19 spike protein to ACE2 is impaired by reduction of the proteins’ disulfide bonds [56]. This was confirmed experimentally by showing that the substitution of Cys488 with alanine in SARS-CoV-2 spike protein impaired pseudotyped SARS-CoV-2 infection, syncytium formation, and cell-cell fusion triggered by SARS-CoV-2 spike expression [57]. Consistently, in vitro binding of ACE2 and RBD, spike-mediated cell-cell fusion, and pseudotyped viral infection of VeroE6/TMPRSS2 cells were inhibited by the thiol-reactive compounds *n*-acetyl-Cys (NAC) and a reduced form of glutathione (GSH), which disrupted the Cys488-S-S-Cys480 disulfide bond in the spike protein.

ACE2 is a tissue enzyme and thus circulating levels are low, however, elevated circulating ACE2 is observed in patients with active COVID-19 disease and in the period after infection [58,59] and has been associated with increased risk of major cardiovascular events [60]. Also, ACE2 serum levels were shown to be significantly elevated in smokers, obese and diabetic individuals [61]. In a study of 306 COVID-19 positive subjects, it has been found that high plasma ACE2 during admission was associated with increased maximal illness severity within 28 days (OR = 1.8, 95%-CI: 1.4–2.3, *p* < 0.0001). Additionally, plasma ACE2 was significantly higher in COVID-19 patients with hypertension compared with patients without hypertension and with pre-existing heart conditions and kidney disease compared with patients without these conditions [62].

Ang II plays its detrimental effect through interaction with angiotensin type 1 receptor (AT1R). AT1R is linked to one-carbon metabolism through Hcy, which has been recently discovered to directly interact and activate AT1R, which aggravates vascular injury. It has been shown that the aggravation of abdominal aortic aneurysm by HHcy is abolished with genetic deletion of AT1 receptor and by blocking of AT1 receptor with telmisartan in animal model. Hcy displaces and limits angiotensin II binding to AT1 receptor. There are distinct conformational changes of AT1 receptor upon binding to angiotensin II and Hcy. It has been suggested that Hcy regulates the conformation of the AT1 receptor by forming a salt bridge and a disulfide bond with its Arg167 and Cys289 residues, respectively. Cys289 of AT1 mediates Hcy-induced AT1 receptor activation. Hcy and angiotensin II synergistically activate the AT1 receptor [63]. It remains to be determined if ACE2 can also be modified by homocysteinylation, leading to the change of its structure/function.

## 9. COVID-19 and Folate Cycle

Apart from activating the glucose metabolism, the SARS-CoV-2 infection activates the folate metabolism. The folate cycle is crucial for the transfer of one-carbon units for nucleotide synthesis. The demand for nucleotide synthesis is increased to match the viral replication needs. Metabolomic studies on Vero E6 cells infected with SARS-CoV-2 have shown that the infection has opposite effects on folate and glutathione abundance, and causes depletion of folate and increase of glutathione level. And interestingly, the elevated glutathione was not crucial for the replication of the virus. However, a drug that is a competitive inhibitor of dihydrofolate reductase and other steps in one-carbon metabolism and nucleotide synthesis, methotrexate, blocks replication and secretion of infectious virions. Experiments with inhibitors of cytosolic and mitochondrial forms of serine hydroxymethyltransferase SHMT1 and SHMT2, respectively), have shown that for virion production particularly important in the cytosolic branch of host one-carbon metabolism, especially for viral subgenomic RNA expression [12].

In one study, plasma folate was significantly reduced in mild (4.7 mg/L, *n* = 74) and severe (4.6 mg/L, *n* = 43) COVID-19 cases compared with healthy controls (12.5 mg/L, *n* = 34) [34]. However, because there were significant differences age and sex between the groups (mild COVID-19: 57.96 years, 52.5% male; severe COVID-19: 71.22 years, 62.8% male; healthy controls (32.65 years, 20.6%) [34], it is not clear whether these differences in folate levels were due to COVID-19 or age/gender differences between the groups.

Some studies have proposed that folic acid might inhibit the binding of the SARS-CoV-2 spike proteins, which blocks the entry of the virus into the cell. One study suggested that vitamin B9 acted as an inhibitor of the furin enzyme, and thus prevented the virus from entering the cell [64]. Another study suggested that folic acid, and its derivatives, 5-methyl tetrahydrofolic acid and tetrahydrofolic acid, have a strong binding affinity against the SARS-CoV-2 [65].

Furin belongs to proprotein convertases family, which cleaves its substrates at Arg-X-X-Arg↓ sites and its impaired activity has been associated with atherosclerosis, cancer and viral infectious diseases. Furin is a ubiquitous endopeptidase, which facilitates SARS-CoV-2 infection by proteolytic cleavage of the spike protein at the S1/S2 cleavage site. This cleavage is essential for entry into human lung cells [66]. Folic acid was tested for the inhibition of furin activity. Docking study results show that folic acid could be an inhibitor of furin and it has been suggested that folic acid could be used in prevention or management of COVID-19-associated respiratory disease in the early stages of the disease [64].

## 10. COVID-19 and Vitamin B_12_

In a small prospective study on forty-nine COVID-19 patients, subjects that had worse condition (subjects admitted to ICU or those that have died, *n* = 9) had significantly higher levels of vitamin B_12_ that those in a better state (*n* = 40) but in a multivariate regression analysis only age was associated with a worse outcome. Folates and Hcy did not differ significantly between the two groups [67]. In another study, levels of vitamin B_12_ did not differ between COVID-19 children and healthy children [24].

## 11. Treatment Strategies Targeting One-Carbon Metabolism

Several possible treatment methods for COVID-19 have been recently discussed [68]. The strategies for therapeutics included polymerase inhibitors, protease inhibitors, interferons, and statins. A variety of nutrients and minerals, such as vitamins A, vitamin B_2_, B_3_, vitamin C, vitamin D, zinc, selenium, and pyrithione have been suggested to be useful in the management of the disease [68]. Additionally, the use of vitamins B_9_, B_12_, probiotics, and magnesium, may also have a positive impact on the prognosis of the infection. Vitamin B_12_ in combination with magnesium and vitamin D has been shown to decrease the severity of COVID-19 [69,70].

Screening hundreds of nutraceuticals compounds against known therapeutic targets of SARS-CoV-2 by molecular docking and the analysis of binding energy have predicted the therapeutic potential of folic acid and its derivatives such as tetrahydrofolic acid and 5-methyl tetrahydrofolic acid against SARS-CoV-2 [65]. Specifically, this computational study found that folic acid was the top nutraceutical predicted to inhibit Spike-ACE-2 interaction, 5-methyltetrahudrofolate bound to PL^pro^, while folic acid derivatives bound to the NSP15 protein. Notably, folates had binding energies that were similar or better than those for known drugs targeting these SARS-CoV-2 proteins.

However, a suggestion that folates could be valuable drugs against COVID-19 [65] appears to be in conflict with findings in Vero E6 cells infected with SARS-CoV-2 showing that antifoliate drugs targeting purine biosynthesis inhibit the virus propagation via antiviral and anti-inflammatory activity [12]. Methotrexate blocks the replication and secretion of infectious virions in the infected Vero cells and may act synergistically with the antiviral nucleotide analog remdesivir, with competes with ATP for incorporation by the viral RNA polymerase [12].

## 12. Conclusions

Multi-omics studies revealed that SARS-CoV-2 infection leads to significant changes in numerous metabolites, including those involved in one-carbon metabolism that impact the virus’s ability to propagate. However, with the exception of three metabolites (glutathione, Met sulfoxide, and choline) that were consistently affected by COVID-19 in various studies, comparative analyses of COVID-19 vs. control samples, both in metabolomics and single compound studies, have often lead to inconsistent results regarding the direction of the change in a particular differentiating metabolite. This could be due to ethnicity differences between various studies as well as age/sex differences between COVID-19 and control groups, which in some studies differed two- to three-fold [21,22,23], (Table S1). This may also be caused by differences in COVID-19 cases classification systems and study design (Table S2), including different sampling times in various studies. In some of the studies, COVID-19 patients are compared to healthy controls; however, taking into account the very diverse symptoms and courses of the illness, this may be an oversimplification. On the other hand, other studies compared groups of subjects manifesting different disease intensity, but are using diverse classification systems, making it difficult to compare the results between different studies. Just how important is the classification system of the COVID-19 course is a study [18], in which, depending on the COVID-19 classification system used, there were or were not differences in SAM levels (Table S2). Finally, data discussed in this review suggest that therapeutic interventions aimed at normalizing glutathione, Met sulfoxide, and choline might provide a promising approach to combat the COVID-19 pandemic. Glutathione can be normalized by supplementation with N-acetyl-Cys [71], or more effectively with Gly + N-acetyl-Cys [28] which, by reducing oxidative stress [28] should also normalize Met sulfoxide. Supplementation with choline can restore its normal levels [72]. Further studies are required to determine the therapeutic potential of targeting these metabolic areas.

## Figures and Tables

**Table 1:** Intracellular metabolites affected by the SARS-CoV-2 infection of Vero E6 cells.

Metabolite	Fold Change ^1^	*p* Value ^2^
Purine biosynthesis		
Folate	0.62	0.0020
5-Formimino-tetrahydrofolate	0.18	0.0018
Serine	0.87	0.0029
Glycine	0.71	0.0025
Ribose-5-Phosphate/Xylulose-5-phosphate	0.91	0.405
5-Phosphoribos-1-pyrophosphate (PRPP)	1.44	0.005
Formylglycinamide ribonucleotide (FGAR)	2.38	2 × 10^−6^
Aminoimidazole ribonucleotide (AIR)	3.10	0.0113
Succinylaminoimidazolecarboxyamide (SAICAR)	1.24	0.0218
Methionine cycle		
Methionine	0.68	0.0020
S-Adenosylmethionine (SAM)	1.01	NS
S-Adenosylhomocysteine (SAH)	1.19	NS
Trans-sulfuration pathway		
Cystathionine	0.70	0.0507
Cysteine	0.80	NS
Glutathione biosynthesis		
Pyroglutamate/5-Oxoproline	0.73	0.0028
Glutathione, reduced (GSH)	1.71	0.0012
Glutathione, oxidized (GSSG)	0.97	NS
Cysteine-glutathione disulfide	0.34	0.0025
Taurine biosynthesis		
Cysteinesulfinic acid	3.10	0.0291
Choline	1.33	0.0014
Betaine	0.78	0.0497

^1^ Recalculated from Supplementary Data 4 in Ref. [12]. ^2^ *t*-test, 2-sided, unequal variance.

**Table 2:** Sulphur metabolites in COVID-19 patients stratified by a degree of lung damage.

Metabolite	Degree of Lung Damage			References
	CT0–1, <5–25% (*n* = 26)	CT2, 26–49% (*n* = 16–18)	CT3–4, 50–75% (*n* = 14–15)	
tGSH, µM	1.81	1.15	1.22 *	[29]
rCG, µM	1.59	1.30	1.29 *	
GSH, µM	1.81	1.15	1.22 ^#^	[18,29]
Hcy, µM	7.4	8.3	9.1	[18]
SAM, nM	59	57	84 ^#^	
SAM/GSH, nM/µM	3.6	7.2 ^&^	5.5	
SAM/GSH, nM/µM	32	57	60 ^#^	

* *p* < 0.05 CT0,1 vs. CT2–4; ^#^ *p* < 0.05 CT3,4 vs. CT0,1; ^&^ *p* < 0.05 CT2 vs. CT0,1. CT, computer tomography.

## Data Availability

Not applicable.

## Supplementary Materials

The following supporting information can be downloaded at: https://www.mdpi.com/article/10.3390/ijms23084181/s1.
