# Supplementary material for: COVID-19 and One-Carbon Metabolism
Source: Int J Mol Sci. 2022 Apr 10;23(8):4181. doi: 10.3390/ijms23084181 (PMC9026976; doi:10.3390/ijms23084181)
Supplement: Supplementary file 1 [file ijms-23-04181-s001.zip › 4-10-22 COVID-19 & OCM, Supplementary Table S1, Table S2.pdf]

COVID-19 and one carbon metabolism

Joanna Perła-Kaján and Hieronim Jakubowski

Supplementary tables

Table S1. Changes in levels of metabolites participating in one-carbon and sulfur amino acid metabolism in COVID-19 patients.

Table S2. Criteria of COVID-19 classifications used in studies of one-carbon and sulfur metabolites.

Table S1. Changes in levels of metabolites participating in one-carbon and sulfur amino acid metabolism in COVID-19 patients.

Metabolite	COVID-19 group classification and size	Median age; % male	Direction of change*	Reference
AICAR	Severe COVID-19, n=23<br>Mild COVID-19, n=14<br>Healthy control, n=17	31; 39%<br>39; 57%<br>50; 47%	Up in serum	Xiao et al. [17]
Adenosine	COVID-19:<br>COVID-19 High IL=6, n=18<br>COVID-19 Medium IL-6, n=10<br>Low IL-6, n=5<br>Healthy control, n=16	57 (mean); 76%<br><br><br><br>38 (mean); 38%	Up in serum	Thomas et al. [23]
	COVID-19, n=139<br>Healthy control, n=133	58; 43%<br>56; 38%	Up in plasma	Su et al. [16]
	Severe COVID-19, n=23<br>Mild COVID-19, n=14<br>Healthy control, n=17	31; 39%<br>39; 57%<br>50; 47%	No change in serum	Xiao et al. [17]
Betaine	COVID-19, n=139<br>Healthy control, n=133	58; 43%<br>56; 38%	No change in plasma	Su et al. [16]
Betaine	Severe COVID-19, n=16<br>Moderate COVID-19, n=16<br>Mild COVID-19, n=20<br>Control, n=9	58; 70%<br><br><br>46; 40%	No change in serum	Caterino et al. [22]
Choline	Mild COVID-19, n=18<br>Healthy control, n=12	7; 72%<br>6; 67%	Down in plasma	Wang et al. [24]
	Severe COVID-19, n=28<br>Non-severe COVID-19, n=37<br>Non-COVID-19, n=25<br>Healthy control, n=28	55; 57%<br>43; 68%<br>53; 68%<br>45; 75%	Down in serum	Shen et al. [43]

Cysteine	Mild COVID-19, n=18<br>Healthy control, n=12	7; 72%<br>6; 67%	Up in plasma	Wang et al. [24]
	Severe COVID-19, n=10<br>Non-severe COVID-19, n=10<br>Healthy control, n=10	65; 70%<br>50; 60%<br>40; 70%	Down in plasma	Li et al. [20]
	Severe COVID-19, n= 46<br>Mild COVID-19, n=19<br>Healthy control, n=27	50 (mean); 63%<br>41 (mean); 53%<br>35 (mean); 37%	Down in serum	Paez-Franco et al. [21]
	Critical, n=28<br>Moderate, n=21<br>Mild, n=23<br>Control, n=27	57; 19%<br>66; 9%<br>40; 9%<br>40; 13%	No change in plasma	Danlos et al. [15]
	Severe COVID-19, n=16<br>Moderate COVID-19, n=16<br>Mild COVID-19, n=20<br>Control, n=9	58; 70%<br><br><br>46; 40%	No change in serum	Caterino et al. [22]
Cysteine-S-sulphate	COVID-19, n=139;<br>Healthy control, n=133	58, 43%<br>56, 38%	Up in plasma	Su et al. [16]
Cystathionine	COVID-19, n=139<br>Healthy control, n=133	58; 43%<br>56, 38%	Up in plasma	Su et al. [16]
Cystathionine	Severe COVID-19, n=23<br>Mild COVID-19, n=14<br>Healthy control, n=17	31;39%<br>39; 57%<br>50; 47%	Up in serum	Xiao et al. [17]
Cystathionine	Mild COVID-19, n=18<br>Healthy control, n=12	7; 72%<br>6; 67%	Down in plasma	Wang et al. [24]
Cystine	Severe COVID-19, n=16<br>Moderate COVID-19, n=16<br>Mild COVID-19, n=20<br>Control, n=9	58; 70%<br><br><br>46; 40%	Up in serum of moderate vs. control	Caterino et al. [22]
	Critical, n=28<br>Moderate, n=21<br>Mild, n=23<br>Control, n=27	57; 19%<br>66; 9%<br>40; 9%<br>40; 13%	Up in plasma, moderate vs. control	Danlos et al. [15]
	COVID-19, n=139<br>Healthy control, n=133	58; 43%<br>56; 38%	Up in plasma	Su et al. [16]
	Severe COVID-19, n= 46<br>Mild COVID-19, n=19<br>Healthy control, n=27	50 (mean); 63%<br>41 (mean); 53%<br>35 (mean); 37%	Up in serum	Paez-Franco et al. [21]
	COVID-19:<br>COVID-19 High IL=6, n=18<br>COVID-19 Medium IL-6, n=10	57 (mean); 76%	Down in serum	Thomas et al. [23]

	Low IL-6, n=5<br>Healthy control, n=16	 38 (mean); 38%		
CysGly	COVID-19, n=139<br>Healthy control, n=133	58; 43%<br>56; 38%	Up in plasma	Su et al. [16]
	CT3,4, n=15<br>CT2, n=18<br>CT0,1, n=26	57; 15%<br>61; 13%<br>65; 18%	Down in plasma	Kryukov et al. [29]
N,N-Dimethylglycine	Mild COVID-19, n=18;<br>Healthy control, n=12	7; 72%<br>6; 67%	Down in plasma	Wang et al. [24]
Glutathione	Mild COVID-19, n=37;<br>Moderate+severe, n=22	63; 27%<br>58; 19%	Down in plasma	Kryukov et al. [29]
	CT3,4, n=15;<br>CT2, n=18;<br>CT0,1, n=26	57; 15%<br>61; 13%<br>65; 18%	Down in plasma	
	COVID-19 patients, n=60:<br>Young adults, n=21;<br>Mid-aged adults, n=21;<br>Older adults, n=18;<br>Healthy controls, n=24<br>(n=8 in each age group)	52 (21-85); 58%<br>21-40;<br>41-60;<br>>60;<br>51;	Down in red blood cells	Kumar P et al. [28]
	Moderate+severe, n=22<br>Mild COVID-19, n=34	57; 19%<br>62; 24%	Down in plasma	Kryukov et al. [18]
	CT3,4, n=14<br>CT2, n=16<br>CT0,1, n=26	56; 14%<br>61; 11%<br>64; 18%	Down in plasma	
	Severe COVID-19, n=23<br>Mild COVID-19, n=14<br>Healthy controls, n=17	31; 39%<br>39; 57%<br>50; 47%	Down in serum	Xiao et al. [17]
Glycine	COVID-19:<br>High IL=6, n=18<br>Medium IL-6, n=10<br>Low IL-6, n=5<br>Healthy control, n=16	57 (mean); 76%<br><br><br><br>38 (mean); 38%	Down in serum	Thomas et al. [23]
	Critical, n=28<br>Moderate, n=21<br>Mild, n=23<br>Control, n=27	57; 19%<br>66; 9%<br>40; 9%<br>40; 13%	Down in plasma of critical vs. control	Danlos et al. [15]
	COVID-19, n=79<br>COVID-19-like, n=30<br>Healthy controls, n=78	51; 60%<br>51; 37%<br>52; 56%	No change in serum	Shi et al. [31]
	COVID-19, n=139<br>Healthy controls, n=133	58; 43%<br>56; 38%	No change in plasma	Su et al. [16]

	Severe COVID-19, n=23<br>Mild COVID-19, n=14<br>Healthy controls, n=17	31; 39%<br>39; 57%<br>50; 47%	No change in serum	Xiao et al. [17]
	Severe COVID-19, n= 46<br>Mild COVID-19, n=19<br>Healthy controls, n=27	50 (mean); 63%<br>41 (mean); 53%<br>35 (mean); 37%	No change in serum	Paez-Franco et al. [21]
	Severe COVID-19, n=16;<br>Moderate COVID-19, n=16;<br>Mild COVID-19, n=20;<br>Control, n=9	58; 70%<br>46; 40%	Up in serum	Caterino et al. [22]
Homocysteine	Severe COVID-19, n=43;<br>Mild COVID-19, n=74;<br>Healthy control, n=34;	71 (mean); 63%<br>58 (mean); 52%<br>33 (mean); 21%	Up in plasma	Keskin et al. [34]
	COVID-19 Survivors, n=270;<br>Non-survivors, n=34	60; 65%<br>73; 65%	Up in plasma of non-survivors	Ponti et al. [39]
	COVID-19 progression group, n=70;<br>COVID-19 progression-free-group, n=202	50; 47%<br>48; 50%	Up in serum	Yang et al. [41]
	Severe COVID-19, n=23;<br>Mild COVID-19, n=14;<br>Healthy control, n=17;	31; 39%<br>39; 57%<br>50; 47%	Down in serum	Xiao et al. [17]
	COVID-19:<br>High IL=6, n=18;<br>Medium IL-6, n=10;<br>Low IL-6, n=5;<br>Healthy control, n=16;	57 (mean); 76%<br>38 (mean); 38%	Down in serum	Thomas et al. [23]
	Moderate+severe, n=22;<br>Mild COVID-19, n=34;	62; 24%<br>57; 19%	No change in plasma	Kryukov et al. [18]
	CT0,1, n=26;<br>CT2, n=16;<br>CT3,4, n=14;	64; 18%<br>61; 11%<br>56; 14%	No change in plasma	
	Severe COVID-19, n=16;<br>Moderate COVID-19, n=16;<br>Mild COVID-19, n=20;<br>Control, n=9	58; 70%<br>46; 40%	No change in serum	Caterino et al. [22]
	Mild COVID-19, n=18;<br>Healthy control, n=12	7; 72%<br>6, 67%	No change in plasma	Wang et al. [24]
Methylmalonic acid	Mild COVID-19, n=18;<br>Healthy control, n=12	7; 72%<br>6, 67%	Down in plasma	Wang et al. [24]

Methionine	COVID-19, n=139;<br>Healthy control, n=133	58; 43%<br>56; 38%	Up in plasma	Su et al. [16]
	Critical, n=28;<br>Moderate, n=21;<br>Mild COVID-19, n=23;<br>Control, n=27	57; 19%<br>66; 9%<br>40; 9%<br>40; 13%	Crit vs. ctrl tended up in plasma<br>Mod. vs. ctrl no change in plasma<br>Mild vs. ctr down in plasma	Danlos et al. [15]
	Severe COVID-19, n= 46;<br>Mild COVID-19, n=19;<br>Healthy control, n=27	50 (mean); 63%<br>41 (mean); 53%<br>35 (mean), 37%	Down in serum	Paez-Franco et al. [21]
	Severe COVID-19, n=10;<br>Non-severe, n=10;<br>Healthy control, n=10	65, 70%<br>50, 60%<br>40, 70%	Down in plasma	Li et al. [20]
	Severe COVID-19, n=16;<br>Moderate COVID-19, n=16;<br>Mild COVID-19, n=20;<br>Control, n=9	58, 70%<br><br><br>46; 40%	No change in serum	Caterino et al. [22]
	Severe COVID-19, n=23;<br>Mild COVID-19, n=14;<br>Healthy control, n=17	31; 39%<br>39; 57%<br>50; 47%	No change in serum	Xiao et al. [17]
Methionine sulphoxide	COVID-19:<br>High IL=6, n=18;<br>Medium IL-6, n=10;<br>Low IL-6, n=5;<br>Healthy control, n=16	57 (mean); 76%<br><br><br><br>38 (mean); 38%	Up in serum	Thomas et al. [23]
	COVID-19, n=139;<br>Healthy control, n=133	58; 43%<br>56; 38%	Up in plasma	Su et al. [16]
	Severe COVID-19, n=23;<br>Mild COVID-19, n=14;<br>Healthy control, n=17	31; 39%<br>39; 57%<br>50; 47%	Up in serum	Xiao et al. [17]
	Mild COVID-19, n=18;<br>Healthy control, n=12	7; 72%<br>6; 67%	Up in plasma	Wang et al. [24]
Methylcysteine	Mild COVID-19, n=18;<br>Healthy control, n=12	7; 72%<br>6; 67%	Down in plasma	Wang et al. [24]
Methylmalonic acid	Mild COVID-19, n=18;<br>Healthy control, n=12	7; 72%<br>6; 67%	Up in plasma	Wang et al. [24]
Methylthioadenosine	COVID-19, n=139;<br>Healthy control, n=133	58; 43%<br>56; 38%	Up in plasma	Su et al. [16]

Pyroglutamate/3-oxoproline	Severe COVID-19, n=23;<br>Mild COVID-19, n=14;<br>Healthy control, n=17	31; 39%<br>39; 57%<br>50; 47%	Down in serum	Xiao et al. [17]
S-Adenosyl-homocysteine	Critical, n=28;<br>Moderate, n=21;<br>Mild, n=23;<br>Control, n=27	57; 19%<br>66; 9%<br>40; 9%<br>40; 13%	Up in plasma	Danlos et al. [15]
	COVID-19, n=139;<br>Healthy control, n=133	58; 43%<br>56; 38%	Up in plasma	Su et al. [16]
	Severe COVID-19, n=23;<br>Mild COVID-19, n=14;<br>Healthy control, n=17	31; 39%<br>39; 57%<br>50; 47%	Down in serum	Xiao et al. [17]
	Moderate+severe, n=22;<br>Mild COVID-19, n=34	57; 19%<br>62; 24%	No change in plasma	Kryukov et al. [18]
	CT3-4, n=14;<br>CT2, n=16;<br>CT0-1, n=26	56; 14%<br>61; 11%<br>64; 18%	No change in plasma	
S-Adenosyl-methionine	Moderate+severe, n=22;<br>Mild COVID-19, n=34	57; 19%<br>62; 24%	No change in plasma	
	CT3-4, n=14;<br>CT2, n=16;<br>CT0-1, n=26	56; 14%<br>61; 11%<br>64; 18%	Up in plasma	
	Severe COVID-19, n=23;<br>Mild COVID-19, n=14;<br>Healthy control, n=17	31; 39%<br>39; 57%<br>50; 47%	No change in serum	Xiao et al. [17]
	Critical, n=28;<br>Moderate, n=21;<br>Mild, n=23;<br>Control, n=27	5; 19%<br>66; 9%<br>40; 9%<br>40; 13%	Up in plasma	Danlos et al. [15]
	Mild, n=71;<br>Severe, n=49	66; 54%<br>69; 65%	Up in serum	Roberts et al. [19]
Serine	COVID-19, n=79;<br>COVID-19 like, n=30;<br>Healthy control, n=78	51; 60%<br>51; 37%<br>52; 56%	Up in serum	Shi et al. [31]
	Non-severe, n=10;<br>Severe COVID-19, n= 10;<br>Healthy control, n=10	65; 70%<br>50; 60%<br>40; 70%	Down in plasma	Li et al. [20]
	COVID-19:<br>Low IL-6, n=5;<br>Medium IL-6, n=10;<br>High IL=6, n=18;<br>Healthy control, n=16	57 (mean); 76%<br>38 (mean); 38%	Down in serum	Thomas et al. [23]

	COVID-19, n=139;<br>Healthy control, n=133	58, 43%<br>56, 38%	No change in plasma	Su et al. [16]
	Severe COVID-19, n=23;<br>Mild COVID-19, n=14;<br>Healthy control, n=17	31; 39%<br>39; 57%<br>50; 47%	No change in serum	Xiao et al. [17]
	Severe COVID-19, n= 46;<br>Mild COVID-19, n=19;<br>Healthy control, n=27	50 (mean); 63%<br>41 (mean), 53%<br>35 (mean), 37%	No change in serum	Paez-Franco et al. [21]
Taurine	COVID-19:<br>High IL=6, n=18;<br>Medium IL-6, n=10;<br>Low IL-6, n=5<br>Healthy control, n=16	57 (mean), 76%<br><br><br><br>38 (mean); 38%	Down in serum	Thomas et al. [23]
	Severe COVID-19, n=23;<br>Mild COVID-19, n=14;<br>Healthy control, n=17	31, 39%<br>39, 57%<br>50, 47%	Down in serum	Xiao et al. [17]
	COVID-19, n=139;<br>Healthy control, n=133	58, 43%<br>56, 38%	No change in plasma	Su et al. [16]
Hypotaurine	COVID-19, n=139;<br>Healthy control, n=133	58, 43%<br>56, 38%	Up in plasma	Su et al. [16]
Vitamin B12	No ICU/alive COVID-19; patients, n=40;<br>ICU or death, n=9	70; 36%<br><br>83, 37%;	Up in plasma of ICU or dead	Dalbeni et al. [67]
	Mild COVID-19, n=18;<br>Healthy control, n=12	7; 72%<br>6, 67%	No change in plasma	Wang et al. [24]

* Assessed from calculations based on the data reported in indicated references and/or the corresponding Supplementary Data files.

**Table S2.** Criteria of COVID-19 classifications	
Classification	Reference
According to the Chinese Government Diagnosis and Treatment Guideline (Trial seventh version), COVID-19 patients were classified into four subgroups based on their different clinical manifestations:<br>(1) mild: mild clinical feature and no pneumonia symptoms;<br>(2) common: fever, respiratory tract symptoms, and imaging features of pneumonia;<br>(3) severe: respiratory distress and respiratory rate ≥30/min OR mean oxygen saturation ≤93% in resting state OR arterial blood oxygen partial pressure (PaO2)/oxygen concentration (FiO2) ≤300 mmHg (1 mmHg=0.133 kPa);<br>(4) critical illness: respiratory failure and require mechanical ventilation OR shock incidence OR require ICU care.<br>In this study, all patients were divided into two subgroups, non-severe (mild and common) and severe (severe and critical illness) cohorts.	Li et al. [20]
The study sample consisted of N = 139 COVID-19 patients (60 males and 79 females) and 133 healthy controls for metabolomics. Enrolled COVID-19 patients have an age range from 18 to 89 (median = 58). For ethnicity, 16 patients were recorded as Hispanic or Latino, 118 were reported as not Hispanic or Latino, and 5 had no ethnicity recorded. For race, 82 patients were recorded as White, 17 patients as Asian, 13 as Black or African American, 4 as Native Hawaiian or other Pacific Islander, 2 as American Indian or Alaska Native, 1 as more than one race, and 20 had no race recorded.	Su et al. [16]
The severity of COVID-19 was evaluated according to the seventh edition of the Guidelines for Diagnosis and Treatment of SARS-CoV-2 issued by the National Health Commission of the People's Republic of China. Severe COVID-19 referred to cases that fulfilled any of the following three criteria: 1) dyspnea, respiratory rate ≥ 30/min; 2) blood oxygen saturation ≤ 93% and ratio of partial pressure of arterial oxygen to fraction of inspired oxygen < 300; and 3) lung infiltrates > 50% within 24 to 48 h. A mild case was defined as a confirmed case with mild symptoms that not fulfilling any of the above criteria. Patients who had symptoms consistent with COVID-19 but were not infected with SARS-CoV-2 were defined as COVID-19-like patients according the abovementioned guidelines and who satisfied the following criteria: 1) fever or respiratory symptoms; 2) imaging manifestations of pneumonia; and 3) optional reduction in white blood cell or lymphocyte count at an early stage. Patients with exposure to COVID-19 individuals only needed to satisfy two of the three criteria for inclusion.	Shi et al. [31]
Severe COVID-19 cases were those meeting any of the following criteria: 1) respiratory distress (≥30 times/min), 2) oxygen saturation ≤ 93% at rest, 3) the arterial partial pressure of oxygen (PaO2)/the fraction of inspired oxygen (FiO2) ≤300 mmHg. Mild patients were defined as COVID-19 patients with	Xiao et al. [17]

symptoms but could not be classified as severe. Symptoms onset date was defined as the date on which symptoms were first observed. Symptoms included fever, fatigue, dry cough, inappetence, myalgia, dyspnea, expectoration, sore throat, diarrhea, nausea, dizziness, headache, abdominal pain, chill, rhinorrhea, chest stuffiness, or nasal congestion.	
COVID-19 patients and control donors were recruited at a third level referral center in Mexico City (Instituto Nacional de Ciencias Médicas y Nutrición Salvador Zubirán) from March to June, 2020. The severity of the disease was classified as follows: Mild/Moderate illness: Fever, signs of airway disease, with or without a tomographic image indicating pneumonia. Severe illness, any of the following: respiratory failure, respiratory rate > 30 bpm, O2 saturation < 92% at rest, PaO2/FiO2 < 300 mmHg52. Once obtained, the serum was processed immediately and frozen to − 80 °C until GC/MS analysis, which was performed	Paez-Franco et al. [21]
The degree of lung damage was then assessed using the following scoring system based on the percentage of lobar involvement: <5% (CT0), 5%–25% (CT1), 26%–49% (CT2), 50%–75% (CT3) and >75% (CT4). Based on the data of an objective study of the respiratory function and blood oxygen saturation, patients were categorized into mild, moderate, and severe groups.	Kryukov et al. [18]
Mild COVID-19 patients (n = 23) were defined by having limited clinical symptoms (fever, cough, diarrhea, myalgia, and anosmia/ageusia) that did not require CT scan or hospitalization. Moderate cases (n = 21) were defined as symptomatic patients with dyspnea and radiological findings of pneumonia on thoracic CT scan, requiring hospitalization and a maximum of 9 L/min of oxygen. Critical patients (n = 28) were those hospitalized in the ICU with respiratory distress requiring 10 L/min of oxygen or more, without or with endotracheal intubation and mechanical ventilation.	Danlos et al. [15]
Severity score metrics based on the 4C Mortality score are provided as group means. Mild 4C score mean=8.39; severe 4C score mean=12.87.	Roberts et al. [19]
The severity of the disease was inferred from serum IL-6 levels, which were determined by a CLIA-certified ELISA-based assay.	Thomas et al. [22]
All COVID-19-children were diagnosed as mild symptoms based on the Diagnosis and Treatment Protocol for Novel Coronavirus Pneumonia (6th edition) of the National Health Commission of China.	Wang et al. [24]
The classification of the COVID-19 cohort was performed on the basis of a seven-point ordinal scale: Patients were classified using an seven-point ordinal scale which consists of following numeric scale and scale descriptors: 1, not hospitalized with resumption of normal activities; 2, not hospitalized, but unable to resume normal activities; 3, hospitalized, not requiring supplemental oxygen; 4, hospitalized, requiring supplemental oxygen; 5, hospitalized, requiring nasal high-flow oxygen therapy, non-invasive mechanical ventilation, or both; 6, hospitalized, requiring ECMO, invasive mechanical ventilation, or both; and 7, death. 1–3 were sub-classified as mild	Caterino et al. [22]

patients, 4 was sub-classified as moderate patients, and 5–7 were sub-classified as severe patients.	
273 diagnosed COVID-19 patients with mild category in Shanghai Public Health Clinical Center were included in this analysis. The primary outcome was defined as imaging progression on chest CT at first week. Any one of the following criteria was considered as imaging progression on chest CT: 1) Increased ground-glass lesions in the underlying involvements; 2) Newly occurred lesions beyond underlying involvements.	Yang et al. [41]
